# 'Surgical cure' for non-parathyroid hypercalcemia

**DOI:** 10.1186/1477-7819-7-23

**Published:** 2009-03-02

**Authors:** Sandeep P Joglekar, Robert L Hudson, Rajesh Logasundaram, Jerome H Pereira

**Affiliations:** 1 James Paget University Hospital NHS Trust, Great Yarmouth, UK; 2 Norfolk and Norwich University Hospital NHS Trust, Norwich, UK

## Abstract

**Background:**

Sarcoidosis is a granulomatous disease of unknown aetiology. Over 90% patients of sarcoidosis present with pulmonary findings. Other organs such as lymph nodes, skin, and joints may be involved. Isolated granulomatous disease confined to the spleen is rare.

**Case presentation:**

This report documents a rare case of isolated granulomatous disease of spleen presenting as hypercalcemia. After all possible causes for hypercalcemia were ruled out, splenectomy was done which proved diagnostic and therapeutic, as calcium levels returned to normal.

**Conclusion:**

We propose that sarcoidosis should be kept in mind as a cause of unexplained hypercalcemia. Increased awareness of radiological features of splenic involvement in sarcoidosis, would help in diagnosis. We believe that we are reporting 9th case in the literature while writing this report.

## Background

Sarcoidosis is an idiopathic multisystem disorder of unknown aetiology which can virtually affect any organ in the body. Splenic involvement is seen in 10–15% patients of which 3% present with palpable spleen. Isolated granulomatous disease confined to spleen is rare. This manifests as multiple splenic nodules which are often difficult to detect on ultrasound scan. Hypercalcemic renal failure is a very rare presentation of isolated splenic involvement, which was seen in our case. Splenic granulomas are the source of calcitriol and splenectomy proves diagnostic as well as therapeutic in such circumstances.

## Case presentation

**Figure 1:**
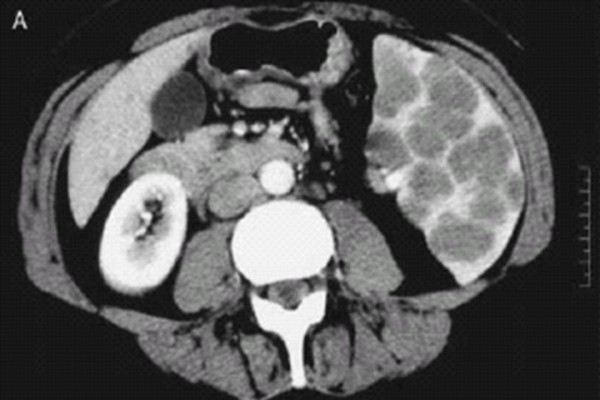
**CT Image – (CT scan image showing multiple splenic nodules)**.

**Figure 2:**
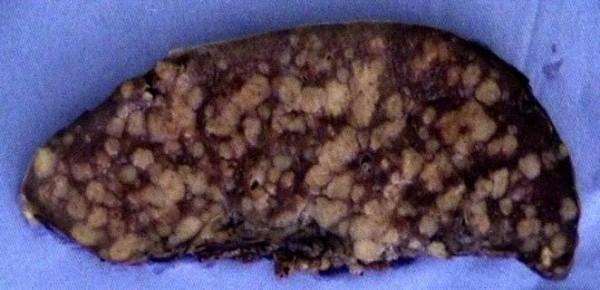
Gross specimen of spleen with the parenchyma studded with circumscribed firm white nodules, which appeared to be confluent in places.

**Figure 3:**
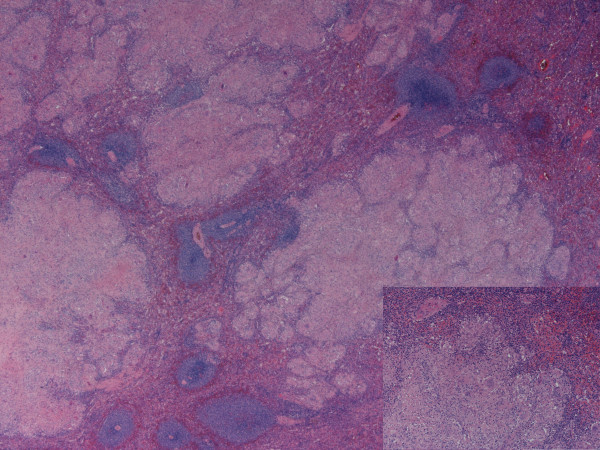
**Photomicrograph showing the nodules to be composed of epithelioid cell granulomas alongwith giant cells (Inset)**.

A 46 year old lady presented with back and leg pain. She had a history of sciatica for 17 years. She also complained of poor appetite, loss of 3 stones of weight in 6 months. Patient also complained of intermittent nausea, vomiting, constipation. Past medical history included previous discectomy and laminectomy, Raynaud's syndrome, essential hypertension, and hysterectomy for endometriosis. Drug allergies included penicillin, erythromycin, septrin, acupan, doxycycline. On examination, pulse and blood pressure were stable. Chest and abdominal examination were normal. Serum investigations revealed a raised corrected calcium of 3.72 mmol/L, urea of 9.4 mmol/L and creatinine of 135 umol/L. ESR was 52 mm/hr, liver function tests were normal. Coagulation studies, protein electrophoresis were within normal limits. Patient subsequently underwent investigations for causes of hypercalcemia. Urine analysis was negative for Bence-Jones proteins. Thyroid function tests were within normal limits. Serum parathormone levels were 1.1 pmol/L. Serum angiotensin convertase enzyme levels were normal (26 U/L). Ultrasound abdomen revealed slightly enlarged spleen. Skeletal survey did not reveal bony secondary deposits or other abnormalities. MRI scan of lumbosacral spine showed posterocentral and right posterolateral disc protrusion at L5/S1 with compression of thecal sac, which explained the back and leg pain. Ultrasound of neck did not reveal thyroid or parathyroid abnormalities. Bone marrow studies were reported normal. CT scan of chest and abdomen was done to rule out occult malignancy as a cause of hypercalcemia. This revealed borderline enlarged spleen (cranio-caudal diameter of 12.7 cms) studded with multiple low density coalescent nodular lesions (figure 1). Patient was started on oral steroids and was given an infusion of alendronate for hypercalcemia. All investigations were discussed in upper GI multidisciplinary meeting and a decision to perform splenectomy was made. Patient underwent laparotomy and splenectomy. Intra-operative findings included enlarged spleen studded with white nodules (figure 2). Postoperative recovery was uneventful. Patient was given chemo-prophylaxis with oral penicillin V and prophylactic pneumococcal and meningococcal vaccines. Histology of spleen showed epitheloid and giant cell granulomas dispersed throughout splenic parenchyma. Granulomas were non-caseating (figure 3). Some of the giant cells within the granulomas contained calcific spherules. Sections from hilar lymph nodes showed a similar granulomatous process. Special stains did not identify fungi or mycobacteria. Final diagnosis of splenic sarcoidosis was made. Postoperatively, calcium levels returned back to normal. Follow-up after one month revealed no further clinical problems and normal serum calcium levels. At 6 months follow up, patient remained asymptomatic. Opthalmic opinion was saught in view of diagnosis of sarcoidosis,. This was found to be entirely normal.

## Discussion

Sarcoidosis is a systemic inflammatory disease of unknown aetiology characterized by the formation of noncaseating granulomas. It occurs most commonly in the third to fifth decades of life [1]. Although sarcoidosis is seen worldwide, the frequency of the disease, organ system involved, acuity of presentation and prognosis vary widely with geography and ethnicity. Over 90% patients with sarcoidosis present with pulmonary findings at the time of diagnosis. Extrapulmonary lesions are seen in liver, eyes, central nervous system, joints and lymph nodes. The reported frequency of splenomegaly in sarcoidosis has ranged from 1% to 40% [2,3]. However, isolated granulomatous disease involving spleen is rare. Primary management consists of medical therapy with prednisolone, methotrexete, and/or anti malarial drugs. Indications for surgery include symptomatic splenomegaly, severe hypersplenism, prophylaxis for splenic rupture, and neoplastic exclusion [4,5]. Our patient presented with symptomatic hypercalcemia and required splenectomy for diagnostic purposes and neoplastic exclusion [6]. The relationship between sarcoidosis and hypercalcemia was first noted in 1932 [7]. Risk factors for development of hypercalcemia in patients with sarcoidosis include renal insufficiency, increased dietary vitamin D, and increased sunlight exposure. Increased bowel absorption caused by a high calcitriol level is the main abnormality [7]. Our patient had hypercalcaemia and elevated urea and creatinine levels. The elevated creatinine is likely due to reversible renal tubular defects (seen in hypercalcemia) causing reduced tubular secretion of creatinine; the interference with renal tubular concentrating function may lead to volume depletion, but our patient was not volume depleted. Abdominal viscera are frequently involved in sarcoidosis, although patients are usually asymptomatic. Liver and spleen are most commonly involved organs, with granulomas noted in 40–60% of patients in two autopsy series. Hypodense splenic nodules are seen in approximately 15% of patients with sarcoidosis [8]. Lesions are usually diffuse. Most nodules are between 0.1 and 3.0 cm, with a mean of approximately 1.0 cm. Isolated or predominant involvement of spleen by nodules is more common than isolated or predominant hepatic nodular disease. Punctate calcifications are relatively uncommon but have been reported as affecting 16% of patients in one study [9]. The occurrence of hepatosplenic nodular sarcoid is more common during first five years of sarcoidosis, with only six of 32 patients in one series having had the disease longer at the time that nodular hepatosplenic sarcoid was diagnosed [10]. Abdominal or systemic symptoms are present in 66% of patients with hepatosplenic sarcoidosis. On contrast-enhanced CT, the splenic nodules are hypodense relative to adjacent normal spleen. Peripheral enhancement is not seen [11]. In one report, lesions visible on contrast-enhanced CT were not seen on sonography, suggesting that the acoustic impedance of the granulomas was similar to that of normal splenic tissue [12]. In our case also, lesions were not seen on ultrasound.

Primary management of splenomegaly in sarcoidosis consists of medical treatment which includes corticosteroids. About 3% of these patients suffer from massive splenomegaly [13,14] resulting in abdominal discomfort, which may be accompanied by thrombocytopenia and other manifestations of hypersplenism [15]. Although patients with splenomegaly respond to corticosteroids given for a long period of time (up to 1 year), most patients with massive splenomegaly will eventually require splenectomy [16]. In our case, patient received initially oral steroids and intravenous infusion of alendronate for hypercalcemia, but the response was only short-lived. Investigations for common causes of hypercalcemia were inconclusive. Our patient had splenectomy for diagnostic purposes and to rule out occult malignancy. Hypercalcemia was successfully treated by splenectomy[17]. Isolated involvement of spleen in sarcoidosis is rare. We believe that at the time of writing this case report, only 8 cases [6,9,18-23] have been so far reported.

## Conclusion

Sarcoidosis as a cause of splenomegaly should be kept in mind. Our case was different from cases so far reported, as our patient presented with hypercalcemia and was successfully treated with splenectomy. Splenectomy not only is helpful for diagnosis but also for treatment of refractory hypercalcemia.

## Consent

Written consent was obtained from the patient for publication of this case report.

## Competing interests

The authors declare that they have no competing interests.

## Authors' contributions

SJ performed the literature search, wrote and submitted the manuscript. RLH assisted with the literature search and obtained the images from the pathology and radiology Dept. RL assisted in pathology reporting and wrote pathology section. JHP performed the surgery, was the consultant in charge of the patients' care and made alterations to the final draft of the paper.

All authors have read and approved final manuscript.
